# The long-term learning curve of holmium laser enucleation of the prostate (HoLEP) in the en-bloc technique: a single surgeon series of 500 consecutive cases

**DOI:** 10.1007/s00345-024-05097-9

**Published:** 2024-07-24

**Authors:** M. J. Wenk, F. O. Hartung, L. Egen, C. Netsch, M. Kosiba, B. Grüne, Jonas Herrmann

**Affiliations:** 1 Department of Urology and Urological Surgery, University Medical Center Mannheim, University of Heidelberg, Theodor-Kutzer-Ufer 1-3, 68167 Mannheim, Germany; 2 Department of Urology, Asklepios Hospital Barmbek, Hamburg, Germany; 3 Department of Urology, University Hospital Frankfurt, Frankfurt Main, Germany

**Keywords:** HoLEP, En-bloc, Prostatic hyperplasia, Learning curve, Endoskopic enucleation, Holmium laser

## Abstract

**Purpose:**

To evaluate perioperative parameters, clinical outcomes, and the learning curve of holmium laser enucleation of the prostate (HoLEP) of a single surgeon in 500 consecutive cases.

**Methods:**

Demographic parameters, outcomes, and adverse events were evaluated. The learning curve for HoLEP in en-bloc technique of the first 500 consecutive patients was analyzed in clusters of 100 (clusters 1–5) using the Wilcoxen rank test, Chi² test and Kruskal Wallis test.

**Results:**

Enucleation weight was similar in the clusters 1,2,3, and 5 (62 g, 63 g, 61 g, 61 g), in cluster 4 it was slightly higher at 73 g. There was a significant reduction in operating time from 67 min (cluster 1) to 57 min (cluster 2), 46 min (cluster 3), 53 min (cluster 4), and 43 min (cluster 5), *p* < 0.001. Enucleation efficiency (g/min) showed a steady increase (1.72, 2.24, 2.79, 2.92 vs. 2.99, *p* < 0.001). Laser energy efficiency also improved (2.17 vs. 2.12 vs. 1.71 vs. 1.65 vs. 1.55; *p* < 0.001). There was no measurable learning curve regarding the length of hospital stay (mean 2.5 days), catheterization time (1.9 days), hemoglobin drop (approx. 1 g/dl) or complications (*p* > 0.1).

**Conclusions:**

HoLEP using the en-bloc technique is a safe and highly effective method. Over time, a slight but steady learning curve and improvement in operation time, enucleation efficiency and laser energy efficiency were shown even for an experienced surgeon - after 500 cases, still no plateau was reached. There was no measurable learning curve regarding blood loss, complications, length of hospital stay, and catheterization time.

## Introduction

While TUR-P was regarded as the gold standard for the surgical treatment of BPH up to a size of 80 cm³ for several decades, endoscopic enucleation techniques are increasingly replacing TUR-P as a size independent alternative [1]. Even though different energy sources for enucleation are associated with different benefits and challenges, several studies have shown that ultimately, technique and surgeon´s experience are more important for the outcome than the energy source itself [2].

For many years, two- and three-lobe techniques with late apical release were the standard methods for enucleation in HoLEP. Lately, en-bloc techniques are gaining increasing acceptance. The en-bloc technique with early apical release as described by Saitta et al. [3] was developed in an effort to reduce the incidence of transient incontinence, a cumbersome side effect that occurs in up to 10% of patients treated with late apical release techniques [4]. In this technique, the sphincter is detached from the adenoma at the start of the procedure, and the mucosa covering the sphincter is carefully preserved in a circumferential manner, thereby reducing mechanical stress during the procedure [5].

The number of cases above which a surgeon can perform HoLEP safely, with satisfactory efficiency and good results can be estimated at 50 cases, requiring a careful case selection [6]. Therefore, the question arises as to what happens after 50 carefully selected cases when the surgeon starts challenging himself with larger glands and less optimal conditions.

To our knowledge, it has not yet been evaluated whether the learning curve for HoLEP stagnates at one point or increases over time, even for very experienced surgeons. Therefore, the aim of this study is to evaluate the learning curve of HoLEP for a surgeon over his first 500 consecutive cases to determine whether further improvements can be expected and whether complication rates are affected.

## Patients and methods

### Study population and data collection

After institutional ethics review board approval (Nr. 2022 − 805), patient data were collected from the hospital information system using the Operation and Procedure Codes for HoLEP. The first 500 consecutive operations that were performed by one surgeon (JH) between January 2021 and September 2023 including his learning curve were evaluated in this study. Bevor switching to HoLEP, the surgeon was experienced in TUR-P (> 100 cases) and ThuLEP (> 50 cases). The ThuLEP cases were performed in three-lobe and two-lobe technique and a few cases in the en-bloc technique. He had not received any specific training prior to starting with HoLEP or the en-bloc technique in particular which was performed in all HoLEP cases from the beginning.

### Parameters

The following preoperative parameters were retrospectively collected from the patients’ electronic medical records: Age, body mass index (BMI), International Prostate Symptom Score (IPSS), IPSS related quality of life (IPSS-QoL), International Index of Erectile Function (IIEF), total prostate specific antigen (PSA), peak urinary flow rate (Q-max), postvoid residual urine volume (PVR), transrectal ultrasound-determined prostate volume (TRUS volume), and oral anticoagulation. In addition, the following parameters enucleation weight, enucleation time, enucleation efficiency (enucleation weight / enucleation time; g/min), operation time, morcellation efficiency (morcellation weight / morcellation time; g/min), hemoglobin loss, laser time efficiency (laser time / weight; min/g), laser energy efficiency (laser energy / weight; kJ/g), length of hospital stay, and the time to catheter removal. Furthermore, transurethral reinterventions and complications according to the Clavien-Dindo classification as well as the Complication Comprehensive Index (CCI) [7] within the first 30 days postoperatively were evaluated.

### Technique and laser settings

HoLEP was performed with the Pulse 120 Moses® Holmium Laser (Lumenis, Yokneam Illit, Israel) with a Slimline® 550 or Moses® 550 Laserfiber. Morcellation was performed using the Piranha® morcellation system in combination with a 26-Fr continuous-flow laser resectoscope (both Richard Wolf, Knittlingen, Germany). The surgeon performed the enucleation in the en-bloc technique with early apical release as described by Saitta [3]. Laser settings for enucleation were 2 J 50 Hz short pulse or Moses Pulse. Coagulation was performed at 1 J and 40 Hz with long pulse setting. Bipolar coagulation was routinely performed in most cases after the enucleation to ensure optimal hemostasis.

### Statistical analysis

Descriptive statistics were carried out to present the baseline and perioperative characteristics. Quantitative data were expressed as mean and standard deviation (SD), and categorical date as absolute and relative frequencies. The cases were analyzed in clusters of 100 (clusters 1–5). The number of clusters was chosen for practical reasons, 5 were chosen corresponding to cluster sizes of 100 patients per cluster. The Kruskal-Wallis test was used to compare perioperative characteristics to different experience levels of the surgeon. Spearman’s correlation analysis was performed to analyze the relationship between the variable of interest and the number of surgical cases. All statistical analyses were carried out with JMP v14 (SAS Institute, Cary, NC, USA). The level of significance was set at 0.05.

## Results

### Baseline characteristics

A total of 500 consecutive patients, who underwent HoLEP at our department between January 2021 und September 2023 and were all operated by the same surgeon, were included in the analyses. Baseline characteristics of the patient cohort are summarized in Table 1. Just over a third of the patients (*n* = 195, 38.7%) were taking an oral anticoagulant preoperatively. All preoperative parameters except for the TRUS volume of the prostate were comparable in the cluster analysis of 100 cases each. For the total cohort of 500 cases, the mean TRUS volume was 93.20 ± 50.36 cm [3].

**Table 1:** Baseline characteristics

Variables	Total (*n* = 500)	Consecutive Cases 1-100	Consecutive Cases 101–200	Consecutive Cases 201–300	Consecutive Cases 301–400	Consecutive Cases 401–500
**Age (year)** Mean (SD)	70.8 (8.48)	69.40 (7.69)	71.18 (8.42)	70.41 (8.76)	71.41 (8.79)	71.29 (8.77)
**BMI (kg/m** ^**2**^ **)** Mean (SD)	27.17 (4.30)	27.37 (4.27)	26.83 (3.97)	27.10 (4.34)	27.22 (4.75)	27.03 (3.75)
**IPSS** Mean (SD)	19.15 (7.46)	21.0 (7.63)	19.01 (7.71)	18.57 (7.16)	18.06 (7.55)	18.77 (6.42)
**IPSS-QoL** Mean (SD)	4.14 (1.51)	4.36 (1.49)	4.01 (1.57)	4.15 (1.60)	4.23 (1.36)	3.81 (1.49)
**IIEF** Mean (SD)	11.06 (8.96)	11.25 (9.43)	12.63 (8.17)	10.5 (8.75)	11.68 (9.39)	9.49 (8.98)
**Total PSA (ng/ml)** Mean (SD)	6.58 (6.73)	6.60 (6.61)	5.92 (5.42)	6.77 (7.88)	6.45 (5.65)	6.91 (7.63)
**Q max (ml/s)** Mean (SD)	9.30 (4.60)	11.60 (4.95)	7.04 (4.13)	7.64 (3.70)	10.28 (3.79)	11.0 (1.83)
**PVR (ml)** Mean (SD)	174.25 (266.60)	217.50 (482.10)	184.23 (167.84)	158.12 (109.87)	156.47 (177.93)	151.62 (147.42)
**TRUS volume (cm**^3^) Mean (SD)	93.20 (50.36)	88.81 (40.28)	87.17 (41.20)	89.3 (46.46)	110.94 (63.07)	87.49 (53.61)
**Oral anticoagulation** n (%)	195 (38.7)	33 (33.3)	43 (43.0)	41 (41.0)	41 (41.0)	36 (36.0)
**Preoperative catheter** n (%)	157 (31.2)	37 (37.4)	18 (18.0)	29 (29.0)	35 (35.0)	36 (36.0)

BMI = body mass index; IPSS = International Prostate Symptom Score; IPSS-QoL = International Prostate Symptom Score related quality of life; IIEF = International Index of Erectile Function; PSA = prostate specific antigen; Q max = peak urinary flow rate; PVR = post-void residual volume; TRUS = transrectal ultrasound, SD = standard deviation

### Perioperative parameters

The perioperative parameters are displayed in Table 2. The mean enucleation weight was 64.68 ± 46.54 g and the time for enucleation was 25.77 ± 16.63 min on average. The mean total operation time was 51.39 ± 28.43 min. Blood loss was low with a mean hemoglobin drop of 0.91 ± 1.04 g/dl. The patients kept their catheters for an average of 1.92 ± 0.89 days and left the hospital after an average of 2.6 ± 1.47 days. The overall complication rate was low at 15.4% (*n* = 77), with the vast majority of complications being minor (Clavien Dindo grade I and II) and no severe complications (Clavien Dindo grade IV and V) occurring. The mean Comprehensive Complication Index was 16.34 ± 9.26. CDC grade I complications (*n* = 39, 7.8%) were urinary retention and prolonged gross hematuria, CDC grade II complications (*n* = 29, 5.8%) were mainly urinary tract infections (*n* = 22, 4.8%) and single cases of anemia, COPD exacerbation, subileus or hypertonia. Two patients needed an endoscopically guided catheter placement due to urinary retention (CDC grade IIIa, 0.4%). CDC grade IIIb complications occurred in seven patients (1.4%), six of them needed reintervention due to bleeding, and one needed a secondary morcellation. Of the six reinterventions due to bleeding or tamponade, three occurred early within 48 h of the surgery and three occurred delayed after discharge from the hospital.

**Table 2:** Perioperative parameters

Variables	Total (*n* = 500)	Consecutive Cases 1-100	Consecutive Cases 101–200	Consecutive Cases 201–300	Consecutive Cases 301–400	Consecutive Cases 401–500
**HoLEP with pulse modulation** n (%)	169 (33.8)	24 (24.2)	7 (7.0)	54 (54.0)	51 (51.0)	29 (29.0)
**Enucleation weight (g)** Mean (SD)	64.68 (46.54)	62.56 (39.75	63.69 (44.09)	61.95 (43.35)	73.31 (56.0)	60.97 (48.39)
**Enucleation time (minutes)** Mean (SD)	25.77 (16.63)	37.59 (25.75)	27.03 (12.61)	20.95 (8.27)	24.43 (12.82)	19.37 (11.09)
**Morcellation time (minutes)** Mean (SD)	14.46 (12.31)	14.37 (10.18)	15.27 (14.91)	13.73 (12.11)	16.43 (12.70)	12.38 (11.21)
**Coagulation time (minutes)** Mean (SD)	7.46 (5.10)	9.52 (4.99)	7.37 (4.36)	6.28 (3.19)	7.34 (4.63)	6.82 (7.12)
**Laser activation time (minutes)** Mean (SD)	17.83 (12.55)	22.54 (9.98)	21.80 (22.52)	16.0 (5.51)	16.07 (7.41)	13.11 (6.91)
**Enucleation efficiency (g/min)** Mean (SD)	2.56 (1.25)	1.73 (0.71)	2.24 (0.93)	2.79 (1.19)	2.93 (1.37)	2.99 (1.38)
**Morcellation efficiency (g/min)** Mean (SD)	4.80 (2.14)	4.73 (1.66)	4.45 (1.39)	4.98 (3.62)	4.68 (1.55)	5.15 (1.65)
**Laser energy efficiency (kJ/g)** Mean (SD)	1.83 (0.99)	2.18 (0.95)	2.13 (1.29)	1.72 (0.73)	1.65 (0.85)	1.55 (0.90)
**Operation time (minutes)** Mean (SD)	51.39 (28.43)	64.05 (26.60)	54.64 (30.77)	45.29 (23.44)	51.24 (28.93)	42.02 (27.75)
**Hemoglobin loss (g/dl)** Mean (SD)	0.91 (1.04)	1.07 (1.04)	0.90 (0.99)	0.68 (1.08)	1.00 (0.92)	0.94 (1.15)
**Length of hospital stay (days)** Mean (SD)	2.61 (1.47)	2.80 (1.67)	2.9 (2.05)	2.56 (1.21)	2.53 (1.14)	2.29 (0.98)
**Time to catheter removal (days)** Mean (SD)	1.92 (0.89)	1.88 (0.95)	1.89 (1.06)	1.74 (0.61)	2.15 (0.98)	1.94 (0.73)
**Blood transfusion** n (%)	2 (0.4)	0 (0)	0 (0)	0 (0)	2 (2.0)	0 (0)
**Postoperative complications**						
**CDC** n (%)						
**CDC Grade I**	39 (7.8)	10 (10.1)	14 (14.0)	5 (5.0)	5 (5.0)	5 (5.0)
**CDC Grade II**	29 (5.8)	4 (4.0)	5 (5.0)	7 (7.0)	8 (8.0)	5 (5.0)
**CDC Grade IIIa**	2 (0.4)	0 (0)	0 (0)	2 (2.0)	0 (0)	0 (0)
**CDC Grade IIIb**	7 (1.4)	0 (0)	2 (2.0)	0 (0)	2 (2.0)	3 (3.0)
**CCI** Mean (SD)	16.50 (9.23)	12.07 (5.79)	13.99 (8.39)	17.30 (6.90)	19.45 (10.08)	21.82 (12.25)

CDC = Clavien Dindo Classification; CCI = Comprehensive Complication Index

### Learning curve

#### Enucleation efficiency

**Fig. 1:**
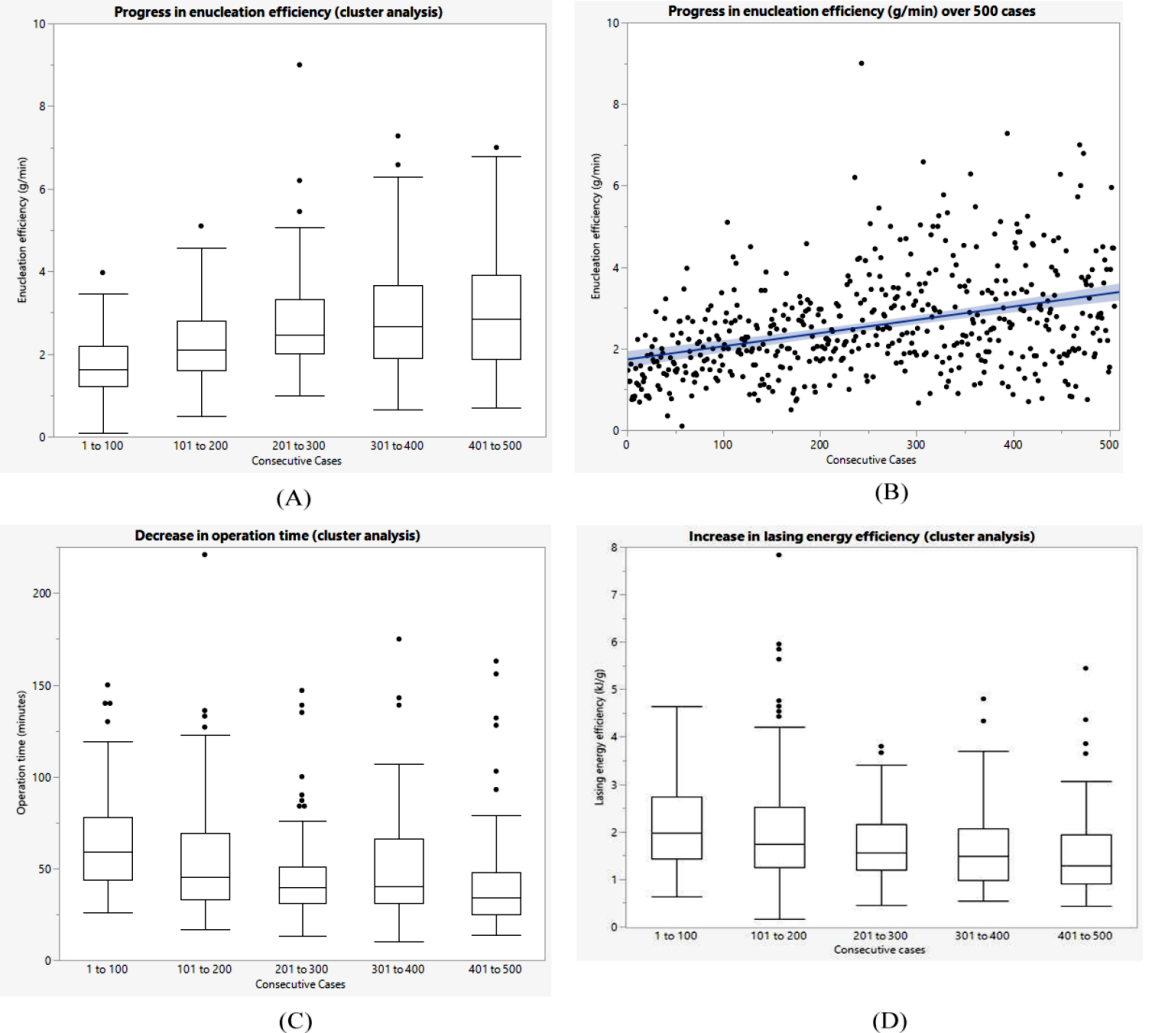
Advancement over 500 cases: (**A**) enucleation efficiency in clusters; (**B**) enucleation efficiency in individual cases; (**C**) operation time in clusters; (**D**) lasing energy efficiency in clusters

The enucleation efficiency increased significantly (Kruskal-Wallis test: *p* < 0.001) from 1.73 ± 0.71 g/min in the first 100 consecutive cases to 2.99 ± 1.38 g/min in the last 100 consecutive cases (cases 401 to 500). The cluster analysis is shown in Fig. 1a using a box plot diagram.

The Spearman rank correlation analysis revealed a statistically significant positive correlation between enucleation efficiency and the number of consecutive cases (*p* < 0.001, ρ = 0.3878) (Fig. 1b).

#### Operation time

The Kruskal-Wallis test showed a significant difference in operation time between the 5 clusters (*p* < 0.001). The operation time decreased from 64.05 ± 26.60 min in the first 100 consecutive cases to 42.02 ± 27.75 min in the last 100 consecutive cases (cases 401 to 500) (Fig. 1c).

#### Lasing energy efficacy

The development of lasing energy efficiency is shown in Fig. 1d. The amount of energy (KJ) used per gram of enucleated tissue decreased significantly, indicating an increase in efficiency (Kruskal-Wallis test: *p* < 0.001) from 2.18 ± 0.95 kJ/g in the first 100 consecutive cases to 1.55 ± 0.90 kJ/g in the last 100 consecutive cases (cases 401 to 500).

## Discussion

Despite anatomical enucleation being recognized for over 50 years as superior to TUR-P in achieving better objective voiding outcomes [8], TUR-P still dominates almost 80% of BPH surgeries in Germany [9] and the US [10]. HoLEP not only surpasses TUR-P in improving BPH symptoms and objective voiding measures such as PVR, flow, and urodynamic parameters, but also reduces risks of bleeding, transfusion, and decreases the catheterization time and hospital stay [11, 12]. A common misconception is the fact that EEP is an option primarily for big glands, since it was seen as a successor of OSP, while in fact it is an excellent choice also for moderate sized glands with substantial advantages over TUR-P [13]. The underutilization of EEP likely stems from its steep learning curve. While its short-term initial learning curve has been well-researched [6, 14, 15], the impact of long-term learning on perioperative outcomes and complications remains unclear. To address this, we analyzed these aspects in a high-volume surgeon’s first 500-cases of en-bloc HoLEP with early apical release.

Throughout the 500 cases, there was a gradual improvement in enucleation time, OR-Time, and enucleation efficiency. The increase in enucleation and OR-Time in Cluster 4 correlates with larger median prostate volumes in this cluster, as enucleation efficiency improved in comparison to Clusters 1,2 and 3. The gradual, linear improvement in enucleation time, despite the consistent use of the same surgical technique, laser, and energy settings, is intriguing. We hypothesize that the key distinction between a novice and an expert lies in the effective use of the instrument for mechanical traction, aiding laser dissection. This balance of mechanical traction and microdissection with the holmium laser, combining visual and tactile input, is inherently complex and seems to get better over time. Data on laser usage supports this: as surgeons use laser energy more effectively, likely helped by improved traction techniques, efficiency increases. Also, the closing gap between laser-activation and enucleation times indicates a more efficient use of both time and energy. Our hypothesis is supported by the findings of Kosiba et al. [16].

Comparing OR times and enucleation efficiency, they were significantly shorter than most surgeons using a two or three lobe technique [17–19], and similar or higher compared to other high volume surgeons using an en-bloc technique [3, 5, 20, 21], highlighting the excellent efficiency of this method [5, 20, 22].

We did not differentiate if pulse modulation was used or not in the evaluation, since a recent study analyzing surgeries of the same surgeon did not show any differences in perioperative outcomes or complications [23].

An encouraging finding from this study is the fact that the OR times and complication rates were short and low in the initial 100 cases already, so surgeons starting HoLEP should not be discouraged: the experience of 500 cases is not necessary to perform a high quality HoLEP, although OR-times will improve over time. Improvements are largely limited to the enucleation phase of the procedure. Looking at the total OR-times and considering turnover times, the improvements are relevant but not substantial. Unsurprisingly, there was no relevant improvement in coagulation- or morcellation times. From the start, complications were infrequent and mostly minor, showing no significant reduction over time. A low rate of bleeding complications requiring reintervention and a relatively minor decrease in hemoglobin levels was observed. This could be attributed to our hybrid coagulation strategy, which combines laser coagulation during the procedure and bipolar coagulation, particularly for mucosal bleeders at the bladder neck, at the end of the enucleation phase.

Although both catheterization time and hospital stay are longer than in most series from the USA [24–28], this is largely a reflection of the reimbursement system rather than the quality of the surgery, therefore, both catheter and hospitalization time is in line with other high volume HoLEP surgeons in Germany [13, 17, 20–22].

This study’s retrospective design is a limitation, as some complications may have been overlooked if patients sought treatment elsewhere, either in other hospitals or outpatient settings. Additionally, being a single-surgeon series, the results reflect one individual’s experience and may not be generalizable to other surgeons. Comorbidities were not included in the analysis. Additionally, the surgeon had experience in ThuLEP before this series, so the initial learning curve should not be compared to surgeons without previous experience in EEP. Despite these constraints, given that the study encompasses 500 consecutive cases over an extended period, it provides valuable insights into the long-term learning curve of HoLEP.

## Conclusion

Over 500 cases, we saw steady improvements in enucleation efficiency, resulting in shorter surgery times, and this improvement continued without a clear limit. The presence of a long-term learning curve may suggest that it might be beneficial to focus training on high-volume HoLEP surgeons.The low rate of complications throughout the study shows that HoLEP is safe, even in the hands of less experienced surgeons – it will just take more time. To sum up, en-bloc HoLEP is a safe and highly effective way to treat BPH. Although there is a long learning curve regarding the OR-time, the quality of the surgery (reflected in outcomes and complication) was not affected by this learning curve.

## References

[1] Naspro R, Gomez Sancha F, Manica M et al (2017) From gold standard resection to reproducible future standard endoscopic enucleation of the prostate: what we know about anatomical enucleation. Minerva Urol Nefrol 69:446 28150483 10.23736/S0393-2249.17.02834-X

[2] Herrmann TR (2016) Enucleation is enucleation is enucleation is enucleation. World J Urol 34:1353 27585786 10.1007/s00345-016-1922-3

[3] Saitta G, Becerra JEA, Álamo D (2019) En Bloc’ HoLEP with early apical release in men with benign prostatic hyperplasia. World J Urol 37:2451 30734073 10.1007/s00345-019-02671-4

[4] Tuccio A, Grosso AA, Sessa F et al (2021) En-Bloc Holmium Laser Enucleation of the prostate with early apical release: are we ready for a New Paradigm? J Endourol 35:1675 33567966 10.1089/end.2020.1189

[5] Rodríguez Socarrás M, Fernández D, Álamo J, Gómez Rivas J, Gómez Sancha F (2020) [En bloc MoLEP (MOSES HoLEP) with early apical dissection and preservation of the sphincter’s mucosa. Surgical technique and technology developments that allow a new paradigm of endoscopic prostate enucleation]. Arch Esp Urol 73:689 33025914

[6] Kampantais S, Dimopoulos P, Tasleem A et al (2018) Assessing the learning curve of Holmium Laser Enucleation of prostate (HoLEP). Syst Rev Urol 120:9 10.1016/j.urology.2018.06.012 30403609

[7] Waldbillig F, Nientiedt M, Kowalewski KF et al (2021) The Comprehensive Complication Index for Advanced Monitoring of complications following endoscopic surgery of the lower urinary tract. J Endourol 35:490 33222525 10.1089/end.2020.0825

[8] Simforoosh N, Abdi H, Kashi AH et al (2010) Open prostatectomy versus transurethral resection of the prostate, where are we standing in the new era? A randomized controlled trial. Urol J 7:262 21170857

[9] Uhlig A, Baunacke M, Groeben C et al (2022) [Contemporary surgical management of benign prostatic obstruction in Germany: a population-wide study based on German hospital quality report data from 2006 to 2019]. Urologe A 61:508 35174398 10.1007/s00120-022-01777-9 PMC9072522

[10] Yin L, Teng J, Huang CJ et al (2013) Holmium laser enucleation of the prostate versus transurethral resection of the prostate: a systematic review and meta-analysis of randomized controlled trials. J Endourol 27:604 23167266 10.1089/end.2012.0505

[11] Pallauf M, Kunit T, Ramesmayer C et al (2021) Endoscopic enucleation of the prostate (EEP). The same but different-a systematic review. World J Urol 39:2383 33956196 10.1007/s00345-021-03705-6 PMC8332586

[12] Wilson LC, Gilling PJ, Williams A et al (2006) A randomised trial comparing holmium laser enucleation versus transurethral resection in the treatment of prostates larger than 40 grams: results at 2 years. Eur Urol 50:569 16704894 10.1016/j.eururo.2006.04.002

[13] Magistro G, Schott M, Keller P et al (2021) Enucleation vs. Resection: a matched-pair analysis of TURP, HoLEP and bipolar TUEP in medium-sized prostates. Urology 154:221 33891930 10.1016/j.urology.2021.04.004

[14] Elzayat EA, Elhilali MM (2007) Holmium Laser Enucleation of the prostate (HoLEP): long-term results, Reoperation Rate, and possible impact of the learning curve. Eur Urol 52:1465 17498867 10.1016/j.eururo.2007.04.074

[15] Enikeev D, Morozov A, Taratkin M et al (2021) Systematic review of the endoscopic enucleation of the prostate learning curve. World J Urol 39:2427 32940737 10.1007/s00345-020-03451-1

[16] Kosiba M, Hoeh B, Welte MN et al (2022) Learning curve and functional outcomes after laser enucleation of the prostate for benign prostate hyperplasia according to surgeon’s caseload. World J Urol 40:3007 36289106 10.1007/s00345-022-04177-y PMC9712403

[17] Deuker M, Rührup J, Karakiewicz PI et al (2021) Holmium laser enucleation of the prostate: efficacy, safety and preoperative management in patients presenting with anticoagulation therapy. World J Urol 39:1219 32488362 10.1007/s00345-020-03272-2 PMC8124040

[18] Kim SC, Matlaga BR, Kuo RL et al (2005) Holmium laser enucleation of the prostate: a comparison of efficiency measures at two institutions. J Endourol 19:555 15989444 10.1089/end.2005.19.555

[19] Kuo RL, Kim SC, Lingeman JE et al (2003) Holmium laser enucleation of prostate (HoLEP): the Methodist Hospital experience with greater than 75 gram enucleations. J Urol 170:149 12796668 10.1097/01.ju.0000070686.56806.a1

[20] Rücker F, Lehrich K, Böhme A et al (2021) A call for HoLEP: en-bloc vs. two-lobe vs. three-lobe. World J Urol 39:2337 33486536 10.1007/s00345-021-03598-5

[21] Miernik A, Schoeb DS (2019) Three horse shoe-like incision holmium laser enucleation of the prostate: first experience with a novel en bloc technique for anatomic transurethral prostatectomy. World J Urol 37:523 30039386 10.1007/s00345-018-2418-0

[22] Tamalunas A, Schott M, Keller P et al (2023) Efficacy, efficiency, and Safety of En-bloc vs three-lobe enucleation of the prostate: a propensity score-matched analysis. Urology 175:48 36828266 10.1016/j.urology.2023.02.014

[23] Hartung FO, Egen L, Grüne B et al. (2024) Pulse modulation in En-Bloc HoLEP: does it really matter? A propensity score matched analysis. World J Urol 42(1): 154 10.1007/s00345-024-04830-8 PMC10940490 38483598

[24] Agarwal DK, Large T, Tong Y et al (2022) Same day discharge is a successful Approach for the majority of patients undergoing Holmium Laser Enucleation of the prostate. Eur Urol Focus 8:228 33414073 10.1016/j.euf.2020.12.018

[25] Agarwal DK, Rivera ME, Nottingham CU et al (2020) Catheter removal on the same day of Holmium Laser Enucleation of the prostate: outcomes of a pilot study. Urology 146:225 33045290 10.1016/j.urology.2020.09.038 PMC7547315

[26] Assmus MA, Large T, Lee MS et al (2021) Same-day discharge following Holmium Laser Enucleation in patients assessed to have large gland prostates (≥ 175 cc). J Endourol 35:1386 33567989 10.1089/end.2020.1218

[27] Assmus MA, Lee MS, Large T, Krambeck AE (2022) Understanding holmium laser enucleation of the prostate (HoLEP) recovery: assessing patient expectations and understanding. Can Urol Assoc J 16:E25 34464254 10.5489/cuaj.7328 PMC8937596

[28] Dean NS, Lee MS, Ganesh M et al (2023) Short-term clinical outcomes of bladder Neck Incision at Time of Holmium Laser Enucleation of the prostate. J Endourol 37:1037 37276153 10.1089/end.2022.0816

